# Therapeutic Management and Prognostic Factors for Ovarian Malignant Tumours in Adolescents: A Comprehensive Review of Current Guidelines

**DOI:** 10.3390/diagnostics13061080

**Published:** 2023-03-13

**Authors:** Chrysoula Margioula-Siarkou, Stamatios Petousis, Georgia Margioula-Siarkou, George Mavromatidis, Fotios Chatzinikolaou, Emmanouel Hatzipantelis, Frédéric Guyon, Konstantinos Dinas

**Affiliations:** 1 2nd Department of Obstetrics and Gynaecology, Aristotle University of Thessaloniki, 541 24 Thessaloniki, Greece; 2 Children’s & Adolescent’s Haematology–Oncology Unit, 2nd Department of Paediatrics, School of Medicine, Aristotle University of Thessaloniki, 541 24 Thessaloniki, Greece; 3 Gynaecologic Oncology Unit, Institut Bergonié, 33000 Bordeaux, France

**Keywords:** ovarian cancer, adolescents, diagnosis, prognosis, treatment, therapy, guidelines

## Abstract

**Background:** Ovarian malignant tumours are rarely diagnosed in adolescents but may have a significant impact on their survival, future fertility and quality of life. The management of such cases is rather complex and requires expertise and careful planning according to scarce existing evidence and recommendations. **Objective:** The aim of this study was to review and compare recommendations from published guidelines regarding the diagnosis, prognosis and treatment of malignant ovarian tumours in adolescents. **Evidence acquisition:** A comparative descriptive/narrative review of guidelines issued by L’Observatoire des Tumeurs Malignes Rares Gynécologiques, the British Society for Paediatric & Adolescent Gynaecology, the European Society for Medical Oncology, the European Society of Gynecological Oncology-European Society for Paediatric Oncology and the European Cooperative Study Group for Pediatric Rare Tumors was conducted. **Results:** All guidelines recommend a thorough diagnostic work-up, consisting of both imaging tests and serum tumour marker measurement, as well as the use of immunohistochemical methods to confirm the diagnosis and complete surgical staging prior to constructing the treatment plan. There is a lack of recommendations regarding the assessment of prognostic factors, with only one guideline providing detailed information. Treatment strategies, as suggested by the majority of guidelines and with only a few discrepancies between them, should include both surgery and adjuvant therapies, mainly chemotherapy, with great emphasis on fertility preservation when it is considered oncologically safe and on the significance of regular and long-term follow-up. **Conclusions:** There is a significant degree of agreement among recommendations of existing guidelines. The reported differences, although limited, highlight the need for the adoption of an international consensus in order to further improve the management of adolescent ovarian cancer.

## 1. Introduction

Ovarian tumours are the most common neoplasms affecting the reproductive system in adolescents [1,2]. They are rarely diagnosed in childhood and adolescence, with an estimated incidence of 2.6 cases per 100,000 girls per year. Approximately 10–30% of all ovarian masses detected in girls up to 17 years are malignant, accounting for 1% of all childhood malignancies and 8% of all abdominal tumours in children [3,4,5]. As expected, due to the particularities of the pediatric population, there are substantial differences between adults and adolescents regarding the incidence, histologic distribution, clinical manifestations, diagnostic evaluation and therapeutic management of ovarian cancer [6,7]. Although epithelial ovarian cancer is the most predominant pathological subtype in adults, ovarian tumours in children and adolescents originate mainly from non-epithelial tissues and cells that are specific to the ovary. Specifically, about 85% of all preadolescent malignant ovarian masses are germ cell tumours (GCTs), 8% are epithelial cell carcinomas, 5% are sex cord stromal tumours (SCSTs) and steroid cell tumours, while less than 1% of them are small cell carcinomas of the ovary [4,8,9]. Germ cell tumours are the most common ovarian neoplasms in pediatric patients, with dysgerminomas and yolk sac tumours being accordingly the most prominent seminomatous and nonseminomatous GCTs in this population [10,11,12]. Regarding sex cord stromal tumours and steroid cell tumours, adolescents are mostly diagnosed with juvenile granulosa cell tumours, Sertoli cell tumours and Sertoli–Leydig cell tumours, rather than granulosa cell tumours and thecomas, which mostly affect peri- and postmenopausal women [9]. The current WHO classification of GCTs and SCSTs are presented in Table 1 [13,14].

The diagnosis of ovarian cancer in the adolescent population can pose challenges in clinical practice, considering the low suspicion of the disease due to young age, the heterogenous and often subtle clinical presentation and the potential limitations in diagnostic imaging in an effort to reduce radiation exposure [15,16]. Ovarian-mass related symptoms are usually non-specific, such as diffuse subacute abdominal and pelvic pain, feeling of pelvic pressure, distended abdomen, rapid increase in abdominal volume, urinary or bowel transit disorders, nausea, vomiting and, much rarer in early puberty, vaginal bleeding and menstrual irregularities [17,18]. Therefore, they can be easily attributed at first in conditions related to other systems rather than the reproductive and, in the majority of cases, the mass is already large at the time of the initial diagnosis [4,19,20]. Moreover, the therapeutic management of ovarian tumours is rather demanding, requiring expertise and precise design, tailored to the specific needs of non-adult patients [21]. Suggested treatment should simultaneously be curative and oncologically safe, ovarian function and fertility-sparing if possible, minimally invasive and sensitive to the psycho–emotional impact on this vulnerable population [1,22,23]. The rarity of ovarian cancer in adolescents further complicates the management of the disease, since the majority of information available to clinicians handling such cases can mostly be obtained by case reports and case series, while only a small number of official guidelines relevant to this topic have been issued. Considering all the above, an effort to combine and concisely summarise all existing guidelines referring to the management of ovarian malignancies in the adolescent population could be very useful to pediatricians, gynecologists and all related specialties involved in such complex cases and it could further facilitate the establishment of evidence-based and generally accepted principals of clinical practice.

The aim of this descriptive review is to compare and synthesise recommendations from published international guidelines regarding the diagnosis, prognosis and treatment of malignant ovarian tumours in adolescents.

## 2. Methods

The main objective of the present narrative review was to identify existing guidelines or recommendations issued by official medical organisations, colleges, associations, societies, committees and study groups regarding the management of ovarian malignant tumours in pediatric and adolescent populations. A search of the literature was conducted in September 2022 through PubMed, Scopus and Web of Science databases. The literature search was performed regarding the period 1990–2022. Electronic search was conducted by using combinations of terms “ovarian cancer” [tiab] OR “ovarian mass”[tiab] AND “adolescent” [tiab] OR “children” [tiab] OR “pediatric” OR “paediatric” [tiab] AND “guidelines” [tiab] OR “recommendations” [tiab]. Additionally, the websites of internationally recognised medical organisations and societies with scientific interest on gynecologic and pediatric oncology were also searched in order to identify official published guidelines relevant to the objective of the present review. Namely, American College of Obstetrics and Gynaecology (ACOG), American Academy of Pediatrics (AAP), American Pediatric Surgical Association (APSA), Society of Obstetricians and Gynaecologists of Canada (SOGC), International Federation of Gynecology and Obstetrics (FIGO), Royal College of Obstetricians and Gynaecologists (RCOG), British Society for Paediatric and Adolescent Gynaecology (BritSPAG), National Institute for Health and Care Excellence (NICE), French Society of Gynaecologic Oncology (SFOG), L’observatoire des tumeurs rares malignes gynécologiques (IMAGYN), European Society of Gynaecologic Oncology (ESGO), European Society of Paediatric Oncology (SIOPE), European Society of Medical Oncology (ESMO), Paediatric Rare Tumours Network-European Registry (PARTNER), Royal Australian and New Zealand College of Obstetricians and Gynaecologists (RANZCOG) and Chinese Society of Obstetrics and Gynaecology (CSOG) were reviewed.

Exclusion criteria included all other types of studies, except for official guidelines and recommendations, as well as guidelines written in any language except for English and French. The main outcomes of interest to identify in the included guidelines/recommendations were the most common types of ovarian cancer in non-adult population, staging and therapeutic management of each type of ovarian cancer in adolescents, as well as suggested follow-up.

Systematic search revealed 96 items with potential for inclusion in our narrative review, of which 12 were duplicated and another 74 were excluded based on title/abstract. There were finally twelve items reviewed as potentially eligible for our review, of which three were excluded because of not reporting guidelines/recommendations, two items were excluded because of not reporting guidelines for paediatric/adolescent population, one item was excluded for reporting only on population with hereditary cancer and one item was excluded because of reporting on population with cancer in pregnancy.

**Figure 1:**
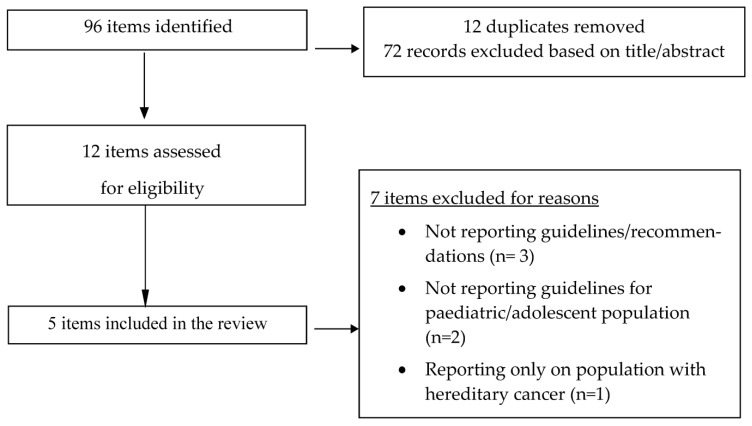
Flowchart of study selection.

There were finally five published guidelines/official recommendations regarding the management of ovarian tumours in children and adolescents that were retrieved and included in the present descriptive/narrative review. In particular, two national guidelines were identified, issued by L’Observatoire des Tumeurs Malignes Rares Gynécologiques (Centres Experts TMRG, 2022) [24] and by the British Society for Paediatric & Adolescent Gynaecology (BritSPAG 2018) [25], as well as two international guidelines, issued by the European Society for Medical Oncology (ESMO 2018) [9], by the European Society of Gynecological Oncology and the European Society for Paediatric Oncology (ESGO-SIOPE 2020) [26] and by the European Cooperative Study Group for Pediatric Rare Tumors as part of the Paediatric Rare Tumours Network-European Registry (EXPeRT/PARTNER 2021) [27,28]. The flowchart of study selection is presented in Figure 1. An overview of recommendations of all five guidelines is presented in Table 2.

## 3. Diagnostic Evaluation

In order to ensure optimal management and due to the rarity of these cases, paediatric and adolescent patients with suspected ovarian malignancies should be referred to a specialised center with a multidisciplinary team composed of trained gynaecological and paediatric oncologists with experience in such cases (ESGO-SIOPE 2020, BritSPAG 2018) [25,26,29,30]. The involvement of a gynaecologist with specialist knowledge of pediatric and adolescent gynaecology is considered necessary, both pre-operatively and post-operatively, even in patients treated for ovarian cysts with low suspicion of malignancy, in terms of supervising the diagnostic and therapeutic management and providing correct counselling about potential future fertility issues (BritSPAG 2018) [25,31].

The preoperative diagnostic work-up should include a combination of tests and modalities, performed to further evaluate suspicious ovarian masses and confirm the diagnosis of ovarian cancer, as well as for staging purposes and the assessment of prognostic factors (ESGO-SIOPE 2020, ESMO 2018, EXPeRT/PARTNER 2021) [9,26,27]. Abdominal and pelvic ultrasound is the initial imaging of choice when investigating ovarian tumours in non-adult patients and it should be performed, if possible, by a gynaecologist or radiologist experienced in paediatric population imaging (Centres Experts TRMG 2022, BritSPAG 2018, EXPeRT/PARTNER 2021) [24,25,27,32]. Transvaginal route of scanning is preferred when the patient is sexually active, while transabdominal scanning is reserved for patients with no sexual relations (BritSPAG 2018) [25], focusing on the pelvis, ovaries, para-aortic lymph nodes (in case of right ovarian tumour) and renal lymph nodes (in case of left ovarian tumour) (EXPeRT/PARTNER 2021) [27]. Evaluation of endometrial thickness should be additionally performed via ultrasound in patients with suspected hormone-producing ovarian tumours, especially SCSTs (ESGO-SIOPE 2020, Centres Experts TMRG 2022) [24,26]. Additional imaging is required, but the recommended imaging tests differ among guidelines; ESGO-SIOPE guidelines suggest that thoracic computed tomography (CT) scan and abdomino–pelvic magnetic resonance imaging (MRI) are necessary, with the latter considered notably useful in assessing bilateral ovarian masses and guiding the choice of surgical approach without exposing the patient to radiation (ESGO-SIOPE 2020, EXPeRT/PARTNER 2021) [26,27,33,34]. On the other hand, an abdomino–pelvic computed tomography (CT) scan and chest X-ray are suggested by ESMO guidelines as additional preoperative imaging tests [9], while guidelines by EXPeRT/PARTNER 2021 recommend chest X-rays for the identification of distant metastases, with the alternative of a low-dose chest CT scan [27]. The use of a positron emission tomography (PET) scan is also debatable, with guidelines by ESMO and L’Observatoire des Tumeurs malignes Rares Gynécologiques underlining that it should be performed in selected patients with suspected germ-cell tumours [9,24], while ESGO-SIOPE guidelines do not support its use due to its low negative predictive value [26]. Preoperative imaging may be omitted in acute settings in favor of immediate surgery (e.g., in clinically suspected ovarian torsion). In that case, the recommended imaging tests should be performed as soon as possible after surgery (ESGO-SIOPE 2020, BritSPAG 2018) [25,26].

There is a consensus among all guidelines that serum tumour markers, especially β-human chorionic gonadotropin (β-hCG), alpha-fetoprotein (AFP), lactate dehydrogenase (LDH) and cancer antigen 125 (CA125), should be measured in all ovarian masses with suspicious features (ESGO-SIOPE 2020, ESMO 2018, BritSPAG 2018, Centres Experts TRMG 2022, EXPeRT/PARTNER 2021) [9,24,25,26,27,35]. A hormonal profile, including oestrogen, testosterone, dehydroepiandrosterone, dehydroepiandrosterone sulfate, luteinising hormone and follicle stimulating hormone levels, is also essential when signs of hormonal production and precocious puberty are identified (BritSPAG 2018, ESGO-SIOPE 2020, EXPeRT/PARTNER 2021) [25,26,27]. Other biomarkers can also be useful; serum calcium, chromogranin A and neuron specific enolase levels can be elevated in small cell carcinoma of the ovary of hypercalcemic type (ESGO-SIOPE 2020, EXPeRT/PARTNER 2021) [26,27], whereas anti-Mullerian hormone (AMH) and inhibin B may indicate the presence of granulosa cell tumours (ESMO 2018, Centres Experts TRMG 2022, EXPeRT/PARTNER 2021) [9,24,27]. Preoperative measurements of tumour markers can provide both diagnostic and prognostic information. In case they are preoperatively elevated, repeated measurements should be performed postoperatively and before the start of adjuvant treatment (for patients receiving adjuvant chemotherapy, new measurements should be obtained before each cycle of treatment) (Centres Experts TRMG 2022, ESGO-SIOPE 2020, ESMO 2018) [9,24,26].

## 4. Pathology and Molecular Biology

A preoperative diagnostic biopsy can definitively provide a histological confirmation of ovarian malignancy, but it should be avoided if a cystic component is identified within the suspicious mass and it is formally indicated only in the case of extraovarian spread of the disease (ESGO-SIOPE 2020, EXPeRT/PARTNER 2021) [26,27]. Tissues retrieved through biopsy and surgical specimens should be examined by an experienced specialist gynaecological or paediatric pathologist, considering that the risk of misdiagnosis is significant due to the rarity of ovarian malignant neoplasms in non-adult patients (ESGO-SIOPE 2020, ESMO 2018, EXPeRT/PARTNER 2021) [9,26,27,36]. The use of immunohistochemistry and molecular tests, if available, is strongly recommended in order to resolve potential diagnostic dilemmas and confirm diagnosis (ESGO-SIOPE 2020) [26]. In suspected germ-cell tumours (GTCs), a panel of immunohistochemical markers including Sall4, OCT3/4, PLAP, NANOG, D2-40, SCFR, a-fetoprotein, glypican-3, SOX2 and SOX10 as well as chromosome 12p fluorescent in situ hybridisation (FISH) for the identification of isochromosome 12 can facilitate the diagnosis in difficult cases. Karyotyping may also be useful, especially in premenarche girls with suspected gonadoblastoma, considering that this type of tumour usually arises in dysgenetic gonads (ESMO 2018, ESGO-SIOPE 2020, Centres Experts TMRG 2022) [9,24,26]. Inhibin A, calretinin, NCAM-1, MART-1, CD99, antigen-like protein 2, steroidogenic factor 1, Forkhead box protein L2, Wilms tumour protein and FOXL2 may be expressed and can be of value in diagnosing sex cord–stromal tumours (SCSTs), especially when they are evaluated in combination, while DICER1 mutations should also be investigated in suspected SCSTs and gynandroblastomas (ESMO 2018, ESGO-SIOPE 2020, Centres Experts TMRG 2022, EXPeRT/PARTNER 2021) [9,24,26,27,37]. Small cell carcinomas of the ovary hypercalcemic type (SCCOHTs) are characterised by the presence of mutations in the SMARCA4 gene, a SWItch/Sucrose Non-Fermentable (SWI/SNF) chromatin-remodelling gene that encodes BRG1 protein. The identification of these mutations, which leads to the loss of BRG1 protein expression, can confirm the diagnosis of SCCOHTs with high sensitivity and specificity (ESMO 2018, ESGO-SIOPE 2020, Centres Experts TRMG 2022) [9,24,26,38,39,40]. Germline mutation analysis and genetic counselling should generally be considered in cases of bilateral GCTs, unilateral GCTs with streak gonad or pubertal retardation, Sertoli–Leydig cell tumour and SCCOHT since these types of tumours can occur as part of a familial tumour syndrome (ESGO-SIOPE 2020) [26].

## 5. Assessment of Prognostic Factors

Although different factors define the prognosis among the various histological types of ovarian cancer in adolescents, the treatment of patients in large specified cancer centers is considered a favourable prognostic factor that applies to all cases (ESMO 2018) [9]. For patients with GCTs, the age of diagnosis can significantly affect the prognosis; premenarche girls may face a worse prognosis than post-adolescent females due to differences in tumour biology. Stage > I, incomplete surgical resection and yolk sac tumour histology are additional adverse prognostic factors for GCTs (ESMO 2018) [9,41,42]. Patients with SCSTs generally have a better prognosis, with 20% of them relapsing or dying from metastatic cancer. Advanced FIGO stage is also recognised as a factor associated with poor outcome for SCSTs along with intraperitoneal tumour rupture and size of tumour > 5 cm (ESMO 2018) [9,35]. On the other hand, prognosis of SCCOHT is poor, given that the percentage of long-time survivors is estimated at 30–40%. The most significant favourable prognostic factors for patients with SCCOHT are stage IA, normal preoperative calcium level, tumour size < 10 cm, absence of large cells and complete surgical resection including bilateral oophorectomy (ESMO 2018) [9,43].

## 6. Surgical Staging

Patients should be staged according to the FIGO 2014 staging system (ESGO-SIOPE 2020, EXPeRT/PARTNER 2021) [26,27]. Surgical staging is of utmost importance to correctly determine the stage of the disease and consequently to further decide on the extent of surgery and the need for postoperative treatment. Regarding a surgical approach, the open route is usually preferred in order to avoid tumour rupture, but a laparoscopic or robotic approach is also acceptable in selected cases (ESMO 2018) [9]. Complete surgical staging includes the sampling of peritoneal fluid before manipulating the tumour or peritoneal washings when no free fluid is detected, careful examination of the abdominal cavity and peritoneal surfaces, biopsy of the diaphragmatic peritoneum, paracolic gutters, pelvic peritoneum, inspection, palpation and large biopsy of the omentum if normal, infracolic omentectomy if omentum is macroscopically abnormal, examination and palpation of pelvic and para-aortic lymph nodes and excision of enlarged ones, inspection of contralateral ovary and biopsy of abnormal appearing areas (ESMO 2018, ESGO-SIOPE 2020, Centres Experts TMRG 2022, EXPeRT/PARTNER 2021) [9,24,26,27]. In case macroscopic disease is detected on other pelvic or abdominal organs, precise description and biopsies of the lesions are also required (ESGO-SIOPE 2020) [26]. For patients with GCTs, systematic ovarian biopsy should be avoided when the non-affected ovary is macroscopically normal. However, in cases with macroscopic bilateral involvement, preservation of a healthy part of one ovary and the uterus should be attempted without compromising oncological safety (ESMO 2018) [9,44]. The role of systematic lymphadenectomy in GCTs in not well-established and should be reserved for cases with nodal abnormalities or residual disease after chemotherapy, considering that nodal recurrence in patients who did not receive initial surgical nodal assessment can be effectively cured with adjuvant chemotherapy (ESMO 2018) [9,45,46]. Early-stage SCSTs rarely produce retroperitoneal or nodal metastases; thus, retroperitoneal evaluation and lymphadenectomy are not mandatory in these cases (ESMO 2018, EXPeRT/PARTNER 2021) [9,27]. On the contrary, extensive peritoneal and nodal surgical staging is indicated in patients with SCCOHT because of the high incidence of extra-ovarian spread (ESMO 2018) [9,47,48].

## 7. General Principles of Therapeutic Management

Although the therapeutic management of ovarian malignances in adolescents is tailored according to the histological type, the stage of the disease and the individual characteristics and needs of each patient, there are some general principles that apply in all cases. Adolescents with non-epithelial ovarian cancer should preferably be treated, when feasible, in the setting of clinical trials (ESGO-SIOPE 2020) [26]. The choice of the surgical approach should be guided by the findings of preoperative imaging and on the basis of avoiding intraoperative tumour rupture. In cases with high suspicion of malignancy, median laparotomy is indicated (ESGO-SIOPE 2020) [26], but in children, a sub-umbilical transverse incision or a Pfannenstiel laparotomy can also be accepted (depending on size of the tumour and the initial tumour spread) (EXPeRT/PARTNER 2021) [27]. On the other hand, a minimally invasive approach is considered an acceptable alternative only if the surgeon is experienced and properly trained in laparoscopic oncological surgery and able to perform a full exploration of the peritoneal cavity and excision of the tumour with no morcellation or accidental rupture (ESGO-SIOPE 2020, EXPeRT/PARTNER 2021) [26,27]. Regarding the radicality of surgical approach, oophorectomy should be, in general, preferred compared to cystectomy or tumourectomy (ESGO-SIOPE 2020, ESMO 2018, EXPeRT/PARTNER 2021) [9,26,27]. However, all efforts should be made to perform, if it is oncologically safe and technically feasible, a fertility sparing surgery at first, with preservation of the uterus and at least a part of one adnexa, considering that saving healthy ovarian tissue is critical both for pubertal development and future fertility (BritSPAG 2018, ESMO 2018, ESGO-SIOPE 2020, EXPeRT/PARTNER 2021) [9,25,26,27,49,50]. A more radical second surgery might be required after the definitive pathological results. In the event of an incidental discovery of a suspicious ovarian tumour during surgery performed by a non-gynaecology specialty for other medical conditions, a gynaecologist should be consulted before attempting surgical manipulations near or on the tumour (BritSPAG 2018) [25].

Oncofertility counselling should be provided to all adolescent patients with ovarian cancer before receiving treatment (ESGO-SIOPE 2020) [26]. Even if ovarian tissue can be preserved during surgery, without compromising the oncological management, there is a significant risk of gonadal dysfunction in patients receiving chemotherapy. The likelihood of chemotherapy-induced amenorrhea depends on the type of administrated drugs, their cumulative dose and the duration of the treatment. Apart from these factors, the age of the patient at the time of chemotherapy also has great impact on the return of menstruation and ovulation, with more favorable results being reported in younger patients (ESMO 2018) [9,51,52]. Taking all the above into consideration, it is evident that oocyte cryopreservation is an option that should be offered to all patients scheduled to receive chemotherapy, either by ovulation induction and oocyte aspiration prior to the beginning of treatment or by controlled ovarian hyperstimulation followed by oocyte cryopreservation 12 months after the end of chemotherapy (ESMO 2018) [9,53,54]. While ovarian stimulation is a safe choice for patients with GCTs, it is permitted only for stage IA granulosa-type SCSTs and after discussion in a multidisciplinary board, while it is contraindicated for stages > IA, in which other fertility preservation techniques that do not require ovarian stimulation should be applied (Centres Experts TMRG 2022) [24]. Fertility and gonadal function preservation is extremely difficult to achieve in patients with SCCOHT, even if one ovary is preserved, due to high-dose combined chemotherapy and radiotherapy that usually follows after surgery (ESMO 2018) [9]. In this setting, it is also crucial to address other acute or delayed side effects of chemotherapy and offer supportive care to minimise adverse symptoms and improve the quality of life of the patients (ESGO-SIOPE 2020) [26].

Young premenopausal patients with GCTs or SCSTs treated with chemotherapy are also eligible for hormone replacement therapy (HRT) in order to relieve the symptoms of potential oestrogen deficiency and iatrogenic menopause, which are often more pronounced compared to those following naturally occurring menopause. HRT can be safely administered to patients with GCTs and SCCOHT, but it should generally be avoided in cases of SCSTs, with the exception of stage IA and IB granulosa-type tumours, where it might be considered upon approval of a multidisciplinary board (ESMO 2018, Centres Experts TMRG 2022) [9,24]. For patients receiving fertility-sparing treatment but wish to postpone pregnancies, hormonal contraception is permitted both in case of a GCT or a SCST, but for the latter, contraceptive products containing oestrogens are contraindicated (ESMO 2018, Centres Experts TMRG 2022) [9,24].

Regular, targeted and long-term follow-up is essential to monitor the response to treatment and to early diagnose a potential recurrence. As already mentioned, the postoperative measurement of serum tumour markers (βhCG, AFP, LDH, CA125, inhibin B) is used to assess tumour response during chemotherapy, in addition to imaging by pelvic ultrasound and CT scan of the abdomen, pelvis and chest, when lung metastases are suspected, while an abdominal MRI could also be useful in case of equivocal findings and patients with poor visibility on ultrasound (ESMO 2018, ESGO-SIOPE 2020, EXPeRT/PARTNER 2021) [9,26,27]. Routine post-treatment monitoring for patients that received fertility-sparing surgery usually includes a pelvic ultrasound every 6 months, whereas a CT scan of the abdomen and pelvis is performed only upon clinical indication. PET scan is not recommended as a tool for follow-up evaluation or tumour response monitoring (ESMO 2018) [9]. The time intervals between each follow-up appointment can vary depending on the histological type of tumour, while discrepancies are also detected between existing guidelines. Apart from clinical care, it is important to continuously offer psycho–oncological support to all patients and their families throughout treatment and follow-up (ESGO-SIOPE 2020) [26].

Specific recommendations for the therapeutic management of germ-cell tumours, sex cord stromal tumours and small cell carcinomas of the ovary will be presented in the following respective sections of the article.

## 8. Germ Cell Tumours (GCTs)

### 8.1. Early Stages

Preoperative MRI is necessary in patients with suspected malignant GTCs with a solid or partially solid mass on ultrasound and if a solid component is also present on MRI; then, surgery is the suggested initial treatment (ESGO-SIOPE 2020) [26]. When planning the surgical approach, it is crucial to assess the likelihood of malignancy according to preoperative imaging. Laparotomy is preferrable for the excision of a GCT with imaging findings suggestive of malignancy because it enables complete surgical staging without increasing the risk of intraperitoneal spillage. During staging, the biopsy of contralateral ovary is not encouraged if it appears to be macroscopically normal (ESGO-SIOPE 2020) [26]. Fertility-sparing surgery should always be offered in adolescent patients with malignant GCTs when it does not jeopardise oncological safety (ESMO 2018, ESGO-SIOPE 2020, Centres Experts TMRG 2022) [9,24,26]. For solid unilateral tumours, total en bloc oophorectomy is recommended, while cystectomy should be avoided in case of a cystic tumour (ESMO 2018, ESGO-SIOPE 2020, Centres Experts TMRG 2022) [9,24,26]. When both ovaries are macroscopically involved, bilateral salpingo-oophorectomy should always be avoided if possible, and maximal effort to preserve at least a part of one ovary and the uterus is encouraged even in stage IB tumours, except when genetic analysis is suggestive of dysgenetic gonads (ESGO-SIOPE 2020, Centres Experts TMRG 2022) [24,26]. Genetic analysis and counselling should be offered to all patients with bilateral ovarian tumours to identify potential sex chromosomal aberrations accompanied by dysgenetic gonads. In that case, bilateral salpingo-oophorectomy is indicated due to the high risk of gonadoblastoma or dysgerminoma (ESGO-SIOPE 2020) [26]. Comprehensive surgical staging with lymphadenectomy should be avoided and reserved only for cases with preoperative or intraoperative evidence of nodal involvement (ESGO-SIOPE 2020) [26].

The need for adjuvant chemotherapy in patients with early-stage GCTs is determined according to the histological type and the stage of the disease. The most used combination is the 5-day bleomycin/etoposide/cisplatin (BEP) regimen (ESMO 2018, Centres Experts TMRG 2022) [9,24]. In stage IA neoplasms where complete surgical resection is achieved, along with normalising or negative post-operative serum tumour markers, active surveillance without adjuvant chemotherapy is usually the approach of choice (ESGO-SIOPE 2020) [26]. Active surveillance involves regular clinical assessment; radiological imaging, including abdominal–pelvic ultrasound CT scan of the abdomen and pelvis, chest X-ray and/or CT scan; and close monitoring of serum tumour marker levels over a period of 10 years, with a gradual increase of intervals between clinical appointments (ESMO 2018, ESGO-SIOPE 2020, Centres Experts TMRG 2022) [9,24,26]. The management of stage IB GCTs is usually more complex and is tailored according to the histotype of the tumours. For stage IC1 GCTs, ESGO-SIOPE guidelines suggest either active surveillance or adjuvant chemotherapy (maximum two cycles), with the latter being the only recommended option for stage IC2-IC3 tumours of any histological type (maximum three cycles) [26], as opposed to the other guidelines which still suggest, as it will be presented below, active surveillance for some IC2-IC3 GCTs. It is worth mentioning that ESGO-SIOPE guidelines [26] are the only official recommendations suggesting two cycles of chemotherapy for stage IC GCTs compared to the guidelines by L’Observatoire des Tumeurs malignes Rares Gynécologiques [24], which recommend two–three cycles of BEP and the guidelines of ESMO [9], which suggest the standard three cycles of chemotherapy, in case the alternative option of close active surveillance (recommended by all three aforementioned guidelines as an acceptable option) is not preferred. The decision both for the administration of chemotherapy instead of surveillance and the duration of the chemotherapy should be carefully made by the multidisciplinary team treating the patient, and the chemotherapy regimen should be tailored individually according to the specific needs of each patient, after taking into consideration the biological behavior and the molecular characteristics of the tumour, since there are slight disparities among guidelines regarding the duration of the regimen.

Active surveillance can be applied for stage IA (and IB, as per guideline by L’Observatoire des Tumeurs malignes Rares Gynécologiques) [24] pure dysgerminomas (ESGO-SIOPE 2020, ESMO 2018) [9,26]. It remains an option for stage IB-IC dysgerminomas with complete surgical resection, with the alternative of adjuvant chemotherapy (ESMO 2018, Centres Experts TMRG 2022) [9,24]. For immature teratomas, active surveillance is recommended for stage IA-IC3 grade 1 tumours (ESMO 2018) [9], whereas it can also be acceptable for IA-IC2 grade 2 tumours (Centres Experts TMRG 2022) [24], for which adjuvant chemotherapy can be alternatively applied (ESMO 2018) [9]. For IA-IC2 grade 3 and IC3 grade 2–3 immature teratomas, adjuvant chemotherapy is usually preferred compared to active surveillance (ESMO 2018, Centres Experts TMRG 2022) [9,24]. Regarding yolk sac tumours, properly staged patients with stage IA (and potentially IB, as suggested by ESMO guidelines) [9] and negative postoperative tumour markers may be monitored with active surveillance instead of receiving adjuvant chemotherapy, which is the sole option for stage IB and IC tumours (ESGO-SIOPE 2020, Centres Experts TMRG 2022) [24,26].

### 8.2. Advanced Stages

Fertility-sparing surgery should be considered even in patients with advanced-stage disease due to the high chemosensitivity of malignant GCTs. For the same reason and given the fact that adjuvant chemotherapy is indicated for all patients with advanced stage GTCs, extensive cytoreductive surgery, which may pose delays in the commencement of postoperative chemotherapy and significantly increase long-term morbidity, should be avoided during initial surgical management (ESMO 2018, ESGO-SIOPE 2020, Centres Experts TMRG 2022) [9,24,26].

Platinum-based chemotherapy agents, mainly the 5-day BEP regimen, remain the treatment of choice for adolescent as well as adult patients. In general, the regimen is administered for three cycles in patients with complete surgical resection and for four cycles in case of macroscopical residual disease, with bleomycin omitted after cycle three to reduce the risk of lung toxicity (ESMO 2018, ESGO-SIOPE 2020) [9,26]. Other regimens that can be used in adolescent population are cisplatine-etoposide-ifosfamide, cisplatin-etoposide-dose-reduced bleomycin or carboplatin-etoposide-bleomycin, all administered for three–four cycles. Patients with elevated serum tumour markers at initial diagnosis who do not have negative markers after cycle four are classified as non-responders to chemotherapy, while if the reduction of marker levels is not the expected, according to their half-life, after cycle two, the patients are identified as high-risk cases in need of potential intensification of the therapy (ESGO-SIOPE 2020) [26]. Moreover, patients already treated with platinum who are diagnosed with a platinum-sensitive relapse (defined as evidence of progression at 4–6 weeks after completion of chemotherapy), the use of combinations with platinum should be considered (ESMO 2018) [9].

A second surgical resection is not always necessary after the completion of chemotherapy. It is recommended in all patients with residual disease (in peritoneum, remaining ovary or lymph nodes) and normal serum cancer markers after chemotherapy, as well as in cases of embryonal carcinomas or non-secreting mixed germ-cell tumours with post-chemotherapy residual lesions. The same applies for immature teratomas, in order to avoid the growing teratoma syndrome (ESMO 2018, ESGO-SIOPE 2020, Centres Experts TMRG 2022) [9,24,26]. However, in patients with immature teratoma and extraovarian spread that comprises gliomatosis peritonei (morphologically benign glial tissue with no immature elements), large and multiple biopsies can be taken instead of complete surgical resection of all lesions (ESGO-SIOPE 2020) [26].

### 8.3. Refractory or Recurrent Disease

When a recurrence of the disease is suspected, a biopsy is necessary to obtain histological confirmation before deciding on additional treatment. It is important to thoroughly examine retrieved specimens in order to identify or rule out the presence of immature tissues. In patients with recurrent disease, normal serum cancer markers and histopathology indicative of an immature teratoma or a mixed tumour with a component of immature teratoma, growing teratoma syndrome should always be included in differential diagnosis. If confirmed, it should be exclusively treated with surgical resection, on the condition of the absence of immature tissues on histological examination (ESGO-SIOPE 2020) [26]. The role of salvage surgery in recurrent disease is not yet established; it is almost exclusively treated with chemotherapy, although there are no well-defined treatment strategies (ESGO-SIOPE 2020, Centres Experts TMRG 2022) [24,26]. Suggested regimens and the duration of therapy depend on the prior administration of chemotherapy. For patients not previously treated with chemotherapy, three or four cycles of BEP (bleomycin, etoposide, cisplatin) should be offered (Centres Experts TMRG 2022) [24]. In patients who have already received chemotherapy, the previous lines of therapy and the time interval between initial diagnosis and relapse should be considered (ESGO-SIOPE 2020) [26]. If BEP regimen was previously administered, patients may benefit from four cycles of either VelP (vinblastine, ifosfamide, cisplatin) or TIP (paclitaxel, ifosfamide, platine) (Centres Experts TMRG 2022) [24]. Intensified chemotherapy with or without stem cell support can be considered when complete response is not achieved (ESGO-SIOPE 2020, Centres Experts TMRG 2022) [24,26]. Finally, for patients with recurrent pure dysgerminoma, the option of radiotherapy could also be discussed (ESGO-SIOPE 2020) [26].

## 9. Sex Cord Stromal Tumours (SCSTs)

### 9.1. Early Stages

Complete surgical staging is imperative in patients with SCSTs, including peritoneal fluid sampling or peritoneal washings, unilateral adnexectomy, examination of contralateral ovary, large omental biopsy or infracolic omentectomy, endometrial curettage for older patients, random blind peritoneal sampling and resection of any suspicious lesions (ESGO-SIOPE 2020, Centres Experts TRMG 2022, EXPeRT/PARTNER 2021) [24,26,27]. Total hysterectomy as part of initial surgery should only be performed in patients with stage II+ disease (Centres Experts TRMG 2022) [24], while omentectomy is recommended only in cases of adhesions to the omentum and not as a routine procedure (EXPeRT/PARTNER 2021) [27]. Systematic lymphadenectomy is not recommended, but the excision of lymph nodes with suspicious preoperative or intraoperative findings is encouraged (ESGO-SIOPE 2020, Centres Experts TRMG 2022, EXPeRT/PARTNER 2021) [24,26,27]. Patients with confirmed Stage IA disease should be treated only with surgery (ESMO 2018, ESGO-SIOPE 2020, Centres Experts TRMG 2022, EXPeRT/PARTNER 2021) [9,24,26,27], during which fertility preservation is feasible as long as all macroscopic lesions are excised (Centres Experts TRMG 2022, EXPeRT/PARTNER 2021) [24,27]. Tumours staged higher than IA (or higher than IB, according to guidelines by EXPeRT/PARTNER) [27] may require chemotherapy (ESGO-SIOPE 2020) [26], which usually consists of three or four cycles of cisplatin-based regimens, mainly BEP, while carboplatin-paclitaxel is also an acceptable option (ESMO 2018, ESGO-SIOPE 2020, Centres Experts TRMG 2022, EXPeRT/PARTNER 2021) [9,24,26,27].

Regarding juvenile granulosa cell tumours, patients with stage IA-IC1 disease and complete surgical resection may avoid chemotherapy, which is otherwise required for stages IC2-IC3 (and potentially for IC1, according to ESMO guidelines (ESMO 2018, ESGO-SIOPE 2020, Centres Experts TRMG 2022 EXPeRT/PARTNER 2021) [9,24,26,27]. For adult granulosa cell tumours, adjuvant chemotherapy is recommended for patients staged as IC3 after complete surgery (and potentially for IC2, as per ESMO guidelines), while it is considered safe to omit it for stage IA-IC2 patients (ESMO 2018, ESGO-SIOPE 2020, Centres Experts TRMG 2022) [9,24,26]. Finally, in completely surgically staged patients with stage IA-IC2 well or moderately differentiated Sertoli–Leydig cell tumours, the omission of chemotherapy is acceptable (Centres Experts TRMG 2022) [24], but it is necessary for poorly differentiated tumours or when heterologous elements/retiform patterns are recognised, even for stage IA cases (ESMO 2018, ESGO-SIOPE 2020) [9,26]. However, guidelines by EXPeRT/PARTNER suggest that adjuvant chemotherapy is recommended in all stage IC Sertoli–Leydig cell tumours, irrespectively of the time of tumour rupture [27].

### 9.2. Advanced Stages

Debulking surgery is considered the most effective course of treatment for advanced stage granulosa cell tumours (ESMO 2018, Centres Experts TRMG 2022) [9,24], although it is considered to have no role in SCSTs except for palliative management, according to EXPeRT/PARTNER guidelines [27]. Adjuvant chemotherapy is generally recommended for patients with advanced-stage SCSTs, preferably with platinum-based combinations. BEP regimen for at least three–four cycles is mainly administered, whereas cisplatin-etoposide-ifosfamide for at least four cycles or carboplatine-paclitaxel for six cycles are alternative options (ESMO 2018, ESGO-SIOPE 2020, Centres Experts TRMG 2022, EXPeRT/PARTNER 2021) [9,24,26,27]. Especially for stage III disease with incomplete initial macroscopic resection and residual disease after chemotherapy, a delayed second surgery after neoadjuvant chemotherapy should be discussed (ESGO-SIOPE 2020, EXPeRT/PARTNER 2021) [26,27].

### 9.3. Refractory or Recurrent Disease

The treatment plan for patients with recurrent SCSTs should be discussed in a multidisciplinary board setting and tailored according to the site of recurrence, histological subtype, dissemination, tumour-free interval and previous therapeutic management. Cytoreductive surgery is the treatment of choice for relapsed patients (ESGO-SIOPE 2020) [26], followed by additional platinum-based chemotherapy (ESMO 2018, Centres Experts TRMG 2022) [9,24]. For patients treated in the setting of a clinical trial, other treatment options may also be available, such as hormone therapy (GnRH agonists, tamoxifen, progestin, aromatase inhibitors) and antiangiogenic and targeted drugs (ESMO 2018, ESGO-SIOPE 2020) [9,26], while the addition of bevacizumab, HIPEC (hyperthermic intraperitoneal chemotherapy with cytoreductive surgery) and regional deep hyperthermia in combination with platinum-based chemotherapy, high-dose chemotherapy with autologous hematopoietic stem cell transplantation and even radiotherapy could be considered on an individual basis for patients with tumour progression and recurrence not responding to other therapeutic options (EXPeRT/PARTNER 2021) [27].

## 10. Small Cell Carcinoma of the Ovary Hypercalcemic Type (SCCOHT)

### 10.1. Early Stages

Confirmation of diagnosis by an expert pathologist and discussion of therapeutic management in a specialised tumour board is suggested due to the rarity of these tumours (ESMO 2018) [9]. Considering treatment strategies, a multimodal approach combining radical surgery, chemotherapy and radiotherapy is recommended. Surgery is always required, including total abdominal hysterectomy, bilateral salpingo-oophorectomy, full pelvic and para-aortic lympadenectomy and peritoneal cytology and peritoneal staging. Due to the aggressive nature of the tumour, a conservative fertility-sparing surgical approach is not oncologically safe. Adjuvant chemotherapy with regimens combining mainly cisplatin and etoposide and potentially paclitaxel is suggested (ESMO 2018, ESGO-SIOPE 2020, Centres Experts TRMG 2022) [9,24,26]. In the absence of evidence of disease after completion of initial chemotherapy, dose-intensive chemotherapy with stem cell support can be applied (ESGO-SIOPE 2020) [26]. The use of pelvic radiotherapy, usually following chemotherapy, may also benefit patients with SCCOHT (ESMO 2018, ESGO-SIOPE 2020, Centres Experts TRMG 2022) [9,24,26].

### 10.2. Advanced Stages

Debulking surgery, either initially or after an interval of three–six cycles of chemotherapy, including omentectomy, systematic pelvic and para-aortic lymph node dissection and complete removal of peritoneal disease is the optional treatment for advanced stage SCCOHT (ESMO 2018, ESGO-SIOPE 2020, Centres experts TRMG 2022) [9,24,26]. Chemotherapy is also indicated with an administration of regimens including platinum and etoposide. When patients achieve complete remission after initial surgery and chemotherapy, they may subsequently be treated with a dose-intensive regimen, followed by high-dose chemotherapy with stem cell support and pelvic radiotherapy (ESGO-SIOPE 2020) [26].

### 10.3. Refractory or Recurrent Disease

Currently, there are no official guidelines about the treatment of patients with recurrent SCCOHT. However, close follow-up is recommended due to the aggressive course and rapid progression of the disease (ESGO-SIOPE 2020) [26].

## 11. Conclusions

Ovarian cancer is rarely diagnosed in children and adolescents, whose diagnostic and therapeutic management can pose great challenges and requires expertise and experience. There is a significant degree of agreement in the recommendations of the existing reviewed guidelines, which generally overlap or complement each other, with only a few areas of dispute. All guidelines suggest that diagnostic work should include both imaging tests (at least ultrasound of abdomen and pelvis) and the measurement of a basic panel of serum tumour markers, while there is debate around the necessity of MRI and the diagnostic value of PET scan. There is a consensus among guidelines about the significant role of molecular biology and immunohistochemistry in confirming the diagnosis, as well as about the procedures that a complete surgical staging should include. On the other hand, only ESMO guidelines provide suggestions about the prognostic factors that should be taken into consideration for adolescent patients with ovarian malignancies. Finally, considering basic therapeutic principles and treatment strategies, recommendations from existing guidelines are mostly identical, suggesting that a combination of fertility-preserving surgery (when it is oncologically safe) and adjuvant therapy is effective in most cases of ovarian cancer in adolescents, with a few discrepancies, mainly detected in proposed time intervals between follow-up appointments. The differences in the reviewed guidelines, although they are limited, highlight the need for the adoption of an international consensus in order to further improve the management of ovarian malignant tumours in the adolescent population.

## Figures and Tables

**Table 1:** WHO (World Health Organisation) 2020 classification of germ cell tumours (GCTs) and sex cord-stromal tumours (SCSTs).

SEX CORD-STROMAL TUMOURS	GERM CELL TUMOURS
**Pure stromal tumours**	Teratoma, benign
Fibroma, NOS	Immature teratoma, NOS
Cellular fibroma	Extra-gonadal teratoma
Thecoma	Post-pubertal type teratoma
Luteinised thecoma associated with sclerosing peritonitis	Dysgerminoma
Sclerosing stromal tumour	Yolk sac tumour
Microcystic stromal tumour	Embryonal carcinoma
Signet ring stromal tumour	Choriocarcinoma, NOS
Leydig cell tumour	Fetus in fetu
Steroid cell tumour	Mixed germ cell tumour
Malignant steroid cell tumour	Monodermal teratomas and somatic type tumours arising from a dermoid cyst
Fibrosarcoma	Struma ovarii, NOS
**Pure sex cord tumours**	Struma ovarii, malignant
Adult granulosa cell tumour	Struma carcinoid
Juvenile granulosa cell tumour	Teratoma with malignant transformation
Sertoli cell tumour, NOS	Cystic teratoma, NOS
Sex cord tumour with annular tubules	Germ cell sex cord stromal tumours
**Mixed sex cord stromal tumours**	Gonadoblastoma
Sertoli–Leydig cell tumour	Dissecting gonadoblastoma
Well differentiated	Undifferentiated gonadal tissue
Moderately differentiated	Mixed germ cell-sec cord stromal tumour, unclassified
Poorly differentiated	
Retiform	
Sex cord stromal tumour, NOS	
Gynandroblastoma	
**Other**	
Papillary cystadenoma	

**Table 2:** Summary of recommendations.

	L’Observatoire des Tumeurs Malignes Rares Gynécologiques (Centres Experts TRMG)	ESGO-SIOPE	ESMO	BritSPAG	EXPeRT/ PARTNER Consensus
Country	France	International/ European	International/ European	United Kingdom	International/ European
Issued	2022	2020	2018	2018	2021
Title	Les tumeurs malignes rares gynécologiques—Référentiels	Non-epithelial ovarian cancers in adolescents and young adults	Non-epithelial ovarian cancer: ESMO Clinical Practice Guidelines for diagnosis, treatment and follow-up	Guideline for the management of ovarian cysts in children and adolescents	Consensus recommendations from the EXPeRT/PARTNER groups for the diagnosis and therapy of sex cord stromal tumours in children and adolescents
**Diagnostic evaluation**					
Reference to specialised centre with multidisciplinary board	Not discussed	Recommended	Not discussed	Recommended	Not discussed
Abdominal–pelvic ultrasound	Recommended as initial imaging	Not discussed	Recommended	Recommended as initial imaging	Recommended
Evaluation of endometrial thickness	Recommended for suspected hormone-producing tumours	Recommended for suspected hormone-producing tumours	Endometrial curettage recommended for adults	Not discussed	Not discussed
Chest X-ray	Not discussed	Not discussed	Recommended	Not discussed	Recommended
CT scan	Not discussed	Recommended thoracic CT scan	Recommended abdominal–pelvic CT scan	Not discussed	Low-dose chest CT as an alternative to chest X-ray
Abdominal–pelvic MRI	Recommended for suspected GCTs	Recommended	Not discussed	Not discussed	Recommended
PET scan	Recommended in selected cases	Not recommended	Recommended in selected cases	Not discussed	Not discussed
Serum tumour markers (basic panel: β-hCG, AFP, LDH, CA125)	Recommended, pre- and post-operative measurement	Recommended, pre- and post-operative measurement	Recommended, pre- and post-operative measurement	Recommended	Recommended
Hormonal profile	Not discussed	Recommended if signs of hormonal production/precocious puberty	Not discussed	Recommended if signs of hormonal production/precocious puberty	Recommended
Post-operative imaging if omitted preoperatively	Not discussed	Recommended	Not discussed	Recommended	Not discussed
**Pathology and molecular biology**					
Preoperative biopsy	Not discussed	Recommended if extraovarian spread, avoided if cystic component	Not discussed	Not discussed	Ovarian biopsy strongly discouraged at diagnosis
Opinion of expert pathologist	Not discussed	Recommended	Recommended	Not discussed	Recommended
Use of immunohistochemical markers	Recommended	Recommended	Recommended	Not discussed	Recommended, FOXL2 for granulosa cell tumours to distinguish adult and juvenile types
Karyotyping	Recommended for suspected gonadoblastoma	Recommended for suspected gonadoblastoma	Recommended for suspected gonadoblastoma	Not discussed	Not discussed
Mutational analysis	Recommended, DICER1 mutations for suspect SCSTs, SMARCA4 mutations for SCCOHT	Recommended, DICER1 mutations for suspect SCSTs, SMARCA4 mutations for SCCOHT, Germline mutation analysis for bilateral GCTs, unilateral GCTs with pubertal retardation, Sertoli–Leydig cell tumours and SCCOHT	Recommended, DICER1 mutations for suspect SCSTs, SMARCA4 mutations for SCCOHT	Not discussed	Recommended, DICER1 mutations for suspected SLCTs or gynandroblastoma
**Assessment of prognostic factors**					
GCTs	Not discussed	Recommended, based on age at diagnosis, FIGO stage, tumour histology, residual disease after surgical resection	Not discussed	Not discussed	Not discussed
SCSTs	Not discussed	Recommended, based on FIGO stage, size of tumour, intraoperative tumour rupture	Not discussed	Not discussed	Not discussed
SCCOHT	Not discussed	Recommended based on FIGO stage, size of tumour, preoperative calcium levels, presence of large cells, residual disease after surgical resection	Not discussed	Not discussed	Not discussed
**Surgical staging**					
Surgical approach	Not discussed	Open route recommended	Not discussed	Not discussed	Open route recommended
Complete surgical staging (peritoneal fluid cytology, complete examination of peritoneal cavity, biopsies of diaphragmatic–paracolic–pelvic peritoneum and abnormal areas, biopsy of omentum, inspection of pelvic–paraaortic lymph nodes and excision of enlarged ones)	Recommended	Recommended	Recommended	Not discussed	Recommended
Biopsy of contralateral ovary	Not discussed	Not recommended if macroscopically normal	Not recommended if macroscopically normal	Not discussed	Not recommended if unsuspicious in palpation and by ultrasound
Systematic pelvic–paraaortic lymphadenectomy	Not discussed	Not routinely recommended	Not routinely recommended, highly indicated for SCCOHT	Not discussed	Not routinely recommended
**General principles of therapeutic management**					
Surgical approach	Not discussed	Median laparotomy recommended when high suspicion of malignancy	Open route recommended	Not discussed	Median laparotomy recommended, sub-umbilical transverse Incision or pfannenstiel laparotomy can be accepted
Tumour resection	Not discussed	Oophorectomy recommended, tumourectomy–cystectomy to be avoided	Unilateral salpingo-oophorectomy recommended	Not discussed	Oophorectomy or adnexectomy is recommended
Fertility-sparing surgery	Not discussed	Recommended if oncologically safe	Recommended if oncologically safe	Recommended if oncologically safe	Recommended for FIGO stage IA SCSTs
Fertility preservation	Ovarian stimulation recommended for GCTs and stage IA granulosa SCSTs	Oncofertility counselling recommended	Oocyte cryopreservation with ovarian stimulation recommended	Not discussed	Not discussed
Hormone replacement therapy	Recommended for GCTs and SCCOHT, to be discussed for IA and IB granulosa SCSTs, not recommended for other SCSTs	Not discussed	Recommended for GCTs and SCCOHT, not recommended for SCSTs	Not discussed	Not discussed
Hormonal contraception (if desired)	Recommended for GCTs, recommended for SCSTs with oestrogen-free products	Not discussed	Recommended for GCTs	Not discussed	Not discussed
Follow-up	Recommended with serum tumour markers, tailored depending on histological type of tumour	Recommended with serum tumour markers, tailored depending on histological type of tumour	Recommended with serum tumour markers, ultrasound and CT scan of abdomen, pelvis ± chest, tailored depending on histological type of tumour	Not discussed	Recommended with serum tumour markers, ultrasound, chest X-ray and abdominal MRI in case of equivocal findings and poor visibility on ultrasound
Supportive care and psycho–oncological support	Not discussed	Recommended	Not discussed	Not discussed	Not discussed
**Treatment of GCTs**				Not discussed	Not discussed
Early stage					
- Surgery	Unilateral oophorectomy recommended	Open unilateral oophorectomy recommended	Unilateral salpingo-oophorectomy recommended		
	For bilateral disease, recommendation for preservation of ovarian tissue if possible	For bilateral disease, recommendation for genetic analysis and preservation of ovarian tissue if possible (bilateral salpingo-oophorectomy recommended if gonadoblastoma or dysgerminoma) Lymphadenectomy only if preoperative/intraoperative evidence of nodal involvement			
- Adjuvant chemotherapy (ChT	BEP is most used regimen	Not recommended for stage IA GCTs with complete surgical resection (only active surveillance needed). Potentially recommended for stage IB GCTs—to be discussed. For stage IC1 GCTs, either ChT or active surveillance recommended For stage IC2-IC3 GCTs, ChT is recommended	BEP is most used regimen		
- Dysgerminomas	Stage IA+IB: active surveillance recommended. Stage IC: active surveillance (if complete surgical resection) or ChT recommended	Stage IA: active surveillance recommended. Stage IB+IC: active surveillance (if complete surgical resection) or ChT recommended.	Stage IA: active surveillance recommended. Stage IB+IC: active surveillance (if complete surgical resection) or ChT recommended.		
- Immature teratomas	Stage IA-IC2 grade 1–2: active surveillance recommended. Stage IA-IC2 grade 3: active surveillance (if complete surgical resection) or ChT recommended. Stage IC3 grade 1: active surveillance recommended. Stage IC3 grade 2–3: active surveillance (if complete surgical resection) or ChT recommended.	Not discussed	Stage IA: active surveillance recommended. Stage IB+IC: active surveillance (if complete surgical resection) or ChT recommended.		
- Yolk sac tumours	Stage IA: active surveillance (if complete surgical resection) or ChT recommended. Stage IB+ IC: ChT recommended.	Stage IA: active surveillance (if complete surgical resection) or ChT recommended. Stage IB+ IC: ChT recommended.	Stage IA+IB: active surveillance (if complete surgical resection) or ChT recommended. Stage IC: ChT recommended.		
Advanced stage					
- Surgery	Fertility sparing surgery to be considered. Second surgery recommended in case of residual disease, immature teratomas, embryonal carcinomas, non-secreting mixed germ-cell tumours with post-chemotherapy residual lesions	Fertility sparing surgery to be considered. Second surgery recommended in case of residual disease and immature teratomas (exception: immature teratoma and gliomatosis peritonei, where large biopsies can instead be taken).	Fertility sparing surgery to be considered. Second surgery recommended in case of residual disease and immature teratomas.		
- Adjuvant chemotherapy (ChT)	BEP regimen is recommended.	BEP regimen for three–four cycles (bleomycin omitted after cycle three) is recommended. Alternative regimens: cisplatine-etoposide-ifosfamide, cisplatin-etoposide-dose-reduced bleomycin, carboplatin-etoposide-bleomycin.	BEP regimen for three–four cycles (bleomycin omitted after cycle three) is recommended. Platinum-sensitive relapse: use of combinations with platinum to be considered.		
Refractory or recurrent disease	Role of surgery unclear, mostly treated with chemotherapy. Prior administration of ChT: BEP (three–four cycles) are recommended. No prior administration of ChT: VelP (vinblastine, ifosfamide, cisplatin) or TIP (paclitaxel, ifosfamide, platine) for three–four cycles to be considered.	Role of surgery unclear, mostly treated with chemotherapy. Prior administration of ChT: previous regimens and the time interval between initial diagnosis and relapse to be considered.	Not discussed		
	Intensified chemotherapy ± stem cell support to be considered in case of incomplete response.	Intensified chemotherapy ± stem cell support to be considered in case of incomplete response. Growing teratoma syndrome with only mature tissues in histology: extensive surgical resection is recommended. Recurrent pure dysgerminoma: radiotherapy to be discussed.			
**Treatment of SCSTs**				Not discussed	
Early stage					
- Surgery	Complete surgical staging (±endometrial curettage) recommended.	Complete surgical staging is recommended.			Complete surgical staging is recommended.
	Total hysterectomy as part only recommended in stage II+.				If adhesions to the omentum, omentectomy is recommended; routine omentectomy not required if unsuspicious
	Lymphadenectomy only if preoperative or intraoperative evidence of nodal involvement.	Lymphadenectomy only if preoperative or intraoperative evidence of nodal involvement.			Routine retroperitoneal lymph node dissection is not recommended if unsuspicious
	Stage IA: only surgery is recommended, fertility preservation acceptable if macroscopic lesions are excised.	Stage IA: only surgery is recommended. Stage IA+: chemotherapy to be considered.	Stage IA: only surgery is recommended.		Stage IA: only surgery is rec-ommended, fertility preservation is acceptable. Stage IA/IB tumours do not require adjuvant ChT if histology shows good to intermediate differentiation.
- Adjuvant chemotherapy (ChT)	BEP (three–four cycles) is recommended, carboplatin-paclitaxel is alternative option.	BEP (three–four cycles) is recommended, carboplatin-paclitaxel is alternative option.	BEP (three–four cycles) is recommended, carboplatin-paclitaxel is alternative option.		ChT protocols include cisplatin-based regimen (e.g., bleomycin–etoposide-cisplatin or etoposide-ifosfamide–cisplatin).
- Juvenile granulosa cell tumours	Stage IA-IC1: ChT may be avoided if complete surgical resection. Stage IC2-IC3: ChT is recommended.	Stage IA-IC1: ChT may be avoided if complete surgical resection. Stage IC2-IC3: ChT is recommended.	Stage IC: ChT is recommended.		In stage IC tumours, three–four cycles of ChT are recommended.
- Adult granulosa cell tumours	Stage IA-IC2: may be avoided if complete surgical resection. Stage IC3: ChT is recommended.		Stage IC2-IC3: ChT is recommended.		Stage IC: ChT is certainly recommended if preoperative spontaneous tumour rupture and/or malignant ascites
- Sertoli–Leydig cells tumours	Stage IA-IC2 well or moderately differentiated Sertoli–Leydig cell tumours: omission of chemotherapy is acceptable.	Stage IC: ChT is recommended.	Stage IA poorly differentiated tumours or with heterologous elements/retiform patterns and Stage >IA: ChT is recommended.		Stage IC: adjuvant ChT is recommended, irrespective of the time of the tumour rupture
Advanced stage					
- Surgery	Debulking surgery: recommended for advanced stages.	Stage III with incomplete initial macroscopic resection and residual disease after chemotherapy: second surgery to be discussed.	Debulking surgery: recommended for advanced stage granulosa cell tumours.		There is no role for debulking surgery (apart from palliative surgery)—inoperable tumours should be biopsied and upfront ChT should be initiated followed by delayed tumour resection.
- Adjuvant chemotherapy (ChT)	Recommended.	Recommended.	Recommended.		In stages II–IV tumours, four cycles of ChT are recommended, with second-look surgery if initial macroscopic incomplete resection or residual disease
	BEP regimen for three–four cycles (alternative option: carboplatine-paclitaxel for six cycles).	BEP regimen for at least four cycles (alternative options: cisplatin-etoposide-ifosfamide for at least four cycles or carboplatine-paclitaxel).	BEP regimen for three cycles (alternative option: carboplatine-paclitaxel for six cycles).		Adjuvant ChT is recommended in all tumours with locoregional spread, distant metastases or unresectable tumours
Refractory/recurrent disease	Platinum-based chemotherapy is recommended.	Treatment plan to be discussed in multidisciplinary board.	Platinum-based chemotherapy is recommended.		
		Cytoreductive surgery recommended treatment of choice for relapsed patients.			
		Additional treatment options to be considered.	Additional treatment options to be considered.		Additional treatment options to be considered.
**Treatment of SCCOHT**					
Early stage				Not discussed	Not discussed
- Surgery	Fertility-sparing surgery is not recommended	Fertility-sparing surgery is not recommended.	Fertility-sparing surgery is not recommended.		
	Radical surgery is recommended (including total abdominal hysterectomy, bilateral salpingo-oophorectomy, full pelvic and para-aortic lympadenectomy)	Radical surgery is recommended (including total abdominal hysterectomy, bilateral salpingo-oophorectomy, full pelvic and para-aortic lympadenectomy).	Radical surgery is recommended (including total abdominal hysterectomy, bilateral salpingo-oophorectomy, full pelvic and para-aortic lympadenectomy)		
- Adjuvant chemotherapy (ChT)	ChT is recommended.	ChT with platinum and etoposide combinations is recommended. Complete remission after initial chemotherapy: dose-intensive chemotherapy with stem cell support to be discussed.	ChT with platinum and etoposide (and potentially paclitaxel) combinations is recommended.		
	Pelvic radiotherapy to be discussed.	Pelvic radiotherapy to be discussed.	Pelvic radiotherapy to be discussed.		
Advanced stage					
- Surgery	Debulking surgery, either initial or interval after three–six cycles of chemotherapy (including systematic pelvic and para-aortic lymphadenectomy) Is recommended.	Debulking surgery, either initial or interval after three–six cycles of chemotherapy (including systematic pelvic and para-aortic lymphadenectomy) is recommended.	Debulking surgery, either initial or interval after three–six cycles of chemotherapy (including systematic pelvic and para-aortic lymphadenectomy) is recommended.		
- Adjuvant chemotherapy (ChT)	ChT is recommended.	ChT with platinum and etoposide combinations is recommended.	ChT with platinum and etoposide combinations is recommended.		
		Complete remission after initial surgery and ChT: dose-intensive regimen, followed by high-dose chemotherapy with stem cell support and pelvic radiotherapy to be considered.			
Refractory/recurrent disease	Not discussed	No suggested treatment.	Not discussed		

AFP: Alpha Fetoprotein; BEP: Bleomycin/Etoposide/Cisplatin; β-hCG: β human chorionic gonadotropin; BritSPAG: British Society for Paediatric & Adolescent Gynaecology; CA125: cancer antigen 125; ChT: Chemotherapy; CT scan: Computerised Tomography scan; ESGO: European Society of Gynecological Oncology; ESMO: European Society for Medical Oncology; EXPeRT: European Cooperative Study Group for Pediatric Rare Tumors; FIGO: International Federation of Gynecology and Obstetrics; GCTs: Germ Cell Tumors; LDH: Lactate Dehydrogenase; MRI: Magnetic Resonance Imaging; PARTNER: Paediatric Rare Tumours Network-European Registry; PET scan: Positron Emission Tomography scan; SCCOHT: Small Cell Carcinomas of the Ovary Hypercalcemic Type; SCSTs: Sex Cord Stromal Tumours; SLCTs: Sertoli–Leydig cell tumours; SIOPE: European Society for Paediatric Oncology; TIP: Paclitaxel/Ifosfamide/Platine; TRMG: Tumeurs Malignes Rares Gynécologiques; VeIP: Vinblastine/Ifosfamide/Cisplatin.
